# The Relationship of Race, Psychosocial Stress and Resiliency Indicators to Neurocognitive Impairment among Older Americans Enrolled in the Health and Retirement Survey: A Cross-Sectional Study

**DOI:** 10.3390/ijerph18031358

**Published:** 2021-02-02

**Authors:** Allan K. Nkwata, Ming Zhang, Xiao Song, Bruno Giordani, Amara E. Ezeamama

**Affiliations:** 1 Department of Epidemiology and Biostatistics, University of Georgia, Athens, GA 30602, USA; mzhang01@uga.edu (M.Z.); xsong@uga.edu (X.S.); 2 Department of Psychiatry, University of Michigan, Ann Arbor, MI 48105, USA; giordani@med.umich.edu; 3 Department of Psychiatry, College of Osteopathic Medicine, Michigan State University, East Lansing, MI 48824, USA

**Keywords:** toxic stress, resilience promoting factors, everyday discrimination, neurocognitive impairment, minority race, older Americans

## Abstract

Background: Race/ethnicity, toxic stress (TS), resilience-promoting factors (RPFs), and their interactions were investigated in relationship to neurocognitive impairment (NI) in a nationally representative sample of adult Americans ≥50 years enrolled in the Health and Retirement Study (HRS) between 2012 and 2014. Methods: NI was defined as physician diagnosis of Alzheimer’s disease/dementia or HRS total cognition score ≤ 10. Race/ethnicity (i.e., African American, White, or Other), TS (i.e., everyday discrimination and chronic stressors), and mastery (as indicator of RPF) were self-reported. Multivariable logistic regression models estimated race-, TS-, RPF-associated odds ratios (ORs), and 95% confidence intervals (CI) for NI adjusting for socio-demographic confounders. Results: 6317 respondents interviewed between the years 2012 and 2014, age range 55–104 years old, 83% White, 13% Black and 4% Other race were included in the study. Chronic stress (OR = 1.88, 95% CI: 1.42–2.48), discrimination (OR = 3.31, 95% CI: 2.12–5.19) and low mastery (OR = 1.85, 95% CI: 1.38–2.48) were each associated with higher NI risk while low mastery was associated with higher NI risk in discrimination and race/ethnicity dependent manner. Specifically, low mastery-associated risk for NI was evident among adults that denied experiencing discrimination (OR = 2.01, 95% CI: 1.51–2.68), but absent among those that experienced discrimination (OR = 0.72, 95% CI: 0.32–1.62). Further, AA race was associated with NI risk but only among adults with high mastery (OR = 2.00, 95% CI: 1.20–3.35). Conclusions: Discrimination, chronic stress, and low mastery were associated with worse cognition. Persisting cognitive disadvantage for AA vs. White/Other race only among high mastery adults suggests that adverse social experiences may counteract mastery-associated cognitive benefits among AA population. TS reduction through policies that promote equal treatment by race/ethnicity in social life, health, justice, and economic systems may promote successful cognitive aging.

## 1. Introduction

As of 2014, an estimated 33% of the United States (US) population self-identified as a member of a racial or ethnic minority group [1]. By the year 2050, an estimated 50% of the US population will be represented by individuals of racial/ethnic minority background [2] such as Hispanic/Latino, Black/African American, Asian/Pacific Islander, and American Indian/Alaska natives.

Despite being a highly diverse country, few aspects of American life are free of racial tension that sometimes manifests as explicit racism—actions guided by the belief that members of one or more races are inferior to members of another race. Racism benefits the political, economic, social, and cultural interests of white Americans at the expense of racial and ethnic minority groups and takes three major forms: institutionalized, personally mediated, and internalized racism [3]. Institutionalized racism is structurally maintained by policies that promote racial inequity in experiences and outcomes in the realm of politics, medicine and access to healthcare, housing, education, employment, and criminal justice systems [4,5]. Personally mediated racism, on the other hand, refers to attitudes and beliefs about the inferiority of minority racial groups (prejudices) and differential treatment of people based on race (discrimination) which is directly experienced at the individual level. Internalized racism refers to the acceptance of negative socio-cultural beliefs about the intrinsic worth of one’s own racial group [6].

Experiences of racial discrimination by minority groups may impact physical and mental health outcomes directly through elevated risk of incarceration, physical injury, patterns of police brutality, or death. Recent data on police-involved fatalities suggests that Black males are 3.2–3.5 times and Hispanic males 1.4–1.7 times more likely to be killed by law enforcement than White males in their lifetime [7,8,9]. More recently, several high-profile cases of police killings of unarmed Black men and women in this country have drawn public attention to the use of lethal force by law enforcement [10]. There is also extensive social justice literature on disparities in the juvenile justice system making the classroom to prison pipeline that is systemically designed to maintain high percentages of African Americans and Hispanics in and out of jail/prison [11,12,13]. Less immediately dangerous but highly prevalent forms of racial discrimination such as microaggressions, lack of respect or courtesy in the workplace, and minority status-related psychosocial stress have been associated with sub-optimal physical [14,15,16] and mental health outcomes [17,18]. High levels of psychosocial stressors that exceed coping resources (resilience-enhancing factors), manifest as toxic stress (TS). Individuals with sufficient coping resources are expected to demonstrate a resilient trajectory and maintain high levels of physical and mental functioning despite high levels of stress-related adversity [19,20]. Resilience promoting factors (RPF) may include personality traits, such as high mastery, or contextual resources, such as social support, that enable individuals to behaviorally resist or down-modulate adverse effects of stressful experiences [21]. TS has been associated with cognitive dysfunction in the domains of learning and memory [22]. Abundant literature documents black–white differences in neurocognitive aging [23,24,25], but specific investigation of TS as a potential mediator or moderator of racial differences in neurocognitive decline or cognitive reserve has not been studied. Further still, although extensive research has demonstrated lifelong sequalae of adverse childhood experiences in older adults [26,27,28], little information is available regarding the potentially mitigating role of RPF as moderators of adverse neurocognitive aging [29,30].

Conditions associated with neurocognitive impairment including Alzheimer’s disease and other forms of dementia are among the leading causes of death in the US [31,32]. The estimated prevalence of dementia among Americans aged ≥ 70 years old in 2010 was 14.7% and is projected to rise with increased life expectancies [33,34]. Additionally, the social and economic costs of dementia among elderly Americans are rising, for instance, in 2010, the total monetary cost of dementia in the US was estimated between $159 and 215 billion [33]. Despite this, we still have a limited understanding of associated risk factors for dementia. Furthermore, given that no clear disease-altering medication is available, we must continue to carefully evaluate risk-associated or risk-lowering environmental and social factors.

This research, grounded in the Stress Process Model (SPM), directly informs present gaps in the understanding of toxic stress (TS)and resilience promoting factors (RPF) as potential mediators or moderators of racial differences in neurocognitive impairment (NI). The SPM postulates that psychosocial resources may impact health directly or indirectly by buffering the negative impacts of stressors [35]. This study specifically examines whether TS and RPF are associated with NI in a nationally representative sample of semi-retired and retired older American adults. The following specific hypotheses are tested: (a) higher levels of TS and lower levels of RPF are associated with higher rates of NI in older American adults, (b) the relationship between race/ethnicity and NI varies according to levels of TS and RPF, and (c) respective relationships between TS, RPF and NI in older adults vary according to race/ethnicity.

## 2. Materials and Methods

We conducted a cross-sectional secondary data analysis of older semi-retired or retired American Adults enrolled in the Health and Retirement Study (HRS) between 2012 and 2014. The HRS is an ongoing biennial study of U.S. adults aged 51 years and older that began in 1992 with the aim of improving our understanding of the social, economic, environmental, and behavioral factors associated with aging and the health of older adults. The study population includes a representative sample of about 20,000 American adults along with their spouses or partners who may be younger than 50 years [36]. Data combines two waves of HRS, that is, HRS 2012 and HRS 2014 as half the sample randomly received the psychosocial leave behind (PLB) questionnaire in each wave. Furthermore, modules that require only a one-time collection such as items on early life trauma, life course stressors, and relationships with parents were omitted from the PLB after 2014 as they have been asked multiple times in previous waves and many new constructs were added [37]. This analysis includes participants with TS, RPF and cognition measures.

### 2.1. Measures

#### 2.1.1. Primary Determinants: Race/Ethnicity, Toxic Stress, and Resilience Promoting Factors

Race/ethnicity was self-reported and categorized as non-Hispanic Black/African American (AA), non-Hispanic White/Caucasian (White) or Other race (i.e., Hispanic or Latino) [38]. Details regarding the items that constitute TS and RPF constructs as well as their scoring processes have been described elsewhere [37]. Briefly, TS was assessed across several domains and included: experiences of everyday discrimination, ongoing chronic stressors, and perceived constraints on personal control [37]. Measures of everyday discrimination (Cronbach’s alpha = 0.83) were six questions that tap into the hassles and chronic stress associated with perceived everyday discrimination and comprised “character assaults” that tend to occur daily (e.g., being treated with less courtesy or respect, receiving poorer service at restaurants and not being thought of as smart). Ongoing chronic stressors (Cronbach’s alpha = 0.67) included eight items that capture current and ongoing problems that have lasted twelve months or longer (e.g., ongoing health problems (in yourself); ongoing physical or emotional problems (in spouse or child) and ongoing problems with alcohol or drug use in a family member). Perceived constraints (Cronbach’s alpha = 0.86) were five items that capture a sense of lack of control of things going on around an individual (e.g., feeling helpless while dealing with the problems of life) [37].

Resilience promoting factors (RPF) on the other hand included global mastery, domain-specific control of finances, health and social life, and measures of social support from spouses, children, relatives, and friends. Global mastery included five questions (Cronbach’s alpha = 0.90) getting at one’s resolve at attaining goals. Domain-specific mastery of health, social life and finances was measured via a single-item measure assessing the amount of control for each aspect on a 10-point scale that ranged from ‘‘no control at all’’ to ‘‘very much control’’, with higher scores indicating greater mastery [37]. Measures of social support included four sets of seven items (Cronbach’s alpha: 0.75–0.86) that examined the level of social support received from spouses/partners, children, other family members and friends. For each relationship category, there were three positively worded items—positive social support (PSS) and four negatively worded items—negative social support (NSS). We created additional constructs to sum up social support responses from immediate family (spouses and children), extended family (friends and other family members), and all four relationship groups (spouses, children, other family members, and friends).

Additionally, for participants with one or two missing items, the score was rescaled to the theoretical maximum for each construct by taking the sum of the items present and dividing it by the number of items present. This quotient was then multiplied by the maximum number of items that could be present. This final number was then rounded to a whole number. All combinations of missing items were accounted for when calculating a score for participants missing two items.

We analyzed TS and RPF scores first as continuous variables and farther dichotomized them based on their distributions. Measures of everyday discrimination were categorized into zero events (reference) and one or more events. Ongoing chronic stressors, perceived constraints, and all RPF were categorized as high vs. low based on the mean distribution of each factor.

#### 2.1.2. Other Measures

Age was assessed by self-reported date of birth and was modeled categorically in 10-year increments. Other covariates included sex, years of education completed, marital status, access to health insurance, body mass index (BMI) and health habits, such as having ever smoked and current alcohol use. The following physician-diagnosed conditions were included in the analysis- heart disease (HD), stroke and Type 2 Diabetes (T2DM), as they are considered important risk factors for neurocognitive impairment and have similar metabolic pathways [39,40].

#### 2.1.3. Outcome

Neurocognitive Impairment (NI) included those with a physician diagnosis of Alzheimer’s disease, Dementia or had a total cognition score ≤10 on the modified Telephone Interview for Cognitive Status (TICSm). TICSm has been described elsewhere [41,42]. Briefly, cognitive functioning measures include immediate and delayed word recall, the serial 7 s test, counting backwards, naming tasks such as date naming, and vocabulary questions. These measures are summed up into three indices; a total recall index for the immediate and delayed word recall tasks, a mental status index for counting, naming and vocabulary tasks, and a total cognition score that sums total recall and mental status indices [43]. Additionally, participants are asked two questions—on physician diagnosis of Alzheimer’s disease, and dementia, senility, or any other serious memory impairment.

### 2.2. Data Analysis

Race, TS, and RPF were analyzed as predictors in relation to presence of NI over two years. First, descriptive analyses determined the distribution of baseline TS, race, RPF, and frequency of NI over 2 years. Bivariate associations were implemented to determine crude associations for NI with race, TS, RPF, potential confounders and sociodemographic factors. Since both TS and RPF were analyzed as categorical variables, chi-square tests were used to evaluate differences in proportion of NI. Factors with a *p*-value ≤ 0.2 were further evaluated in multivariable models as candidate confounders. Multivariable logistic regression models were used to estimate odds ratios (ORs) and 95% confidence intervals (CIs) with adjustment for candidate confounders such as age, sex, education, alcohol consumption, and comorbidity due to diabetes, heart diseases, and stroke. A series of incremental nested models were implemented, beginning with crude models, followed by models with sociodemographic factors adjusted for, and models adjusting for sociodemographic factors as well as TS. The final models further adjusted for RPF. Additionally, separate regression models evaluated interaction between race/ethnicity and respective TS and RPF and the potential for interaction between TS and RPF. *P*-values for interaction effects were set at *p* < 0.10 because the power of statistical tests for higher order terms is generally lower than for first-order terms [44,45]. When potential interactions were indicated, stratum specific results were presented. For all multivariable analyses, missing confounder values were addressed using listwise deletion so that the analytic sample included all participants with a complete set of data on variables specified. All results were adjusted for the complex sampling design of the HRS [46]. All analyses were implemented with SAS software, version 9.4 (SAS Institute, Cary, NC, USA).

## 3. Results

Included were 6317 respondents interviewed between the years 2012 and 2014 and ranged in age from 55 and 104 years of age. The analytic sample included 83% non-Hispanic White, 13% Black/African American, and 4% Other race (includes Hispanics). Majority of the sample were female (60%), 62% were married/partnered, 46% had some college education or more, 50% had a cardiometabolic diagnosis (HD, T2DM, or stroke), and about 5% were neurocognitively impaired. Of note nearly half (49%) reported ≥ three comorbid conditions. (Table 1).

Across race groups, the proportions of individuals reporting TS varied significantly at baseline for experiences of everyday discrimination (*p* < 0.0001), chronic stressors (*p* = 0.008) and personal constraints (*p* = 0.0009) and were higher amongst minority groups. Similarly, the prevalence of NI was higher among minority groups relative to Whites (*p* = 0.04, Table 2). Sociodemographic factors, BMI, and lifestyle factors measured at baseline by race are reported in Supplementary Table S1.

### 3.1. Race Is Not Associated with Cognitive Impairment, But Disparities Persist According to Level of Mastery

**Figure 1:**
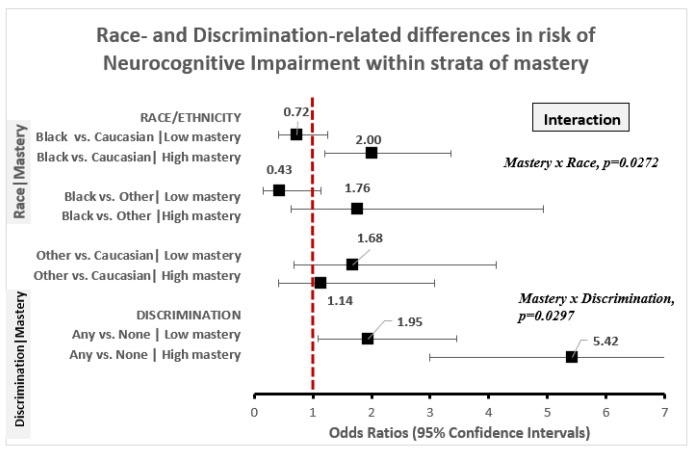
Race and discrimination-related differences in risk of Neurocognitive impairment within strata of mastery.

Unadjusted for confounding covariates, the odds of NI were 76% elevated (OR = 1.76, 95% CI: 1.21–2.56) Black vs. White Americans. However, this association was not robust to adjustment for socio-demographic confounders, comorbidity, and resiliency indicators (Table 3). However, race-related differences in risk of NI were dependent on the level of mastery among older Americans (mastery x race, *p* = 0.027; Figure 1). On one hand, among Americans with high mastery, African American race was associated with 100% increased odds of NI, relative to Caucasian race (OR = 2.00, 95% CI: 1.20–3.35), and with 76% increased odds of NI relative to Other race (OR = 1.76, 95% CI: 0.63–4.93), but the odds of NI was similar for those that identified as Other relative to Caucasian race (OR = 1.14, 95% CI: 0.42–3.07). On the other hand, among American adults with low mastery, race-related differences in odds of NI were absent. Specifically, African American race was on average associated with lower NI odds relative to Caucasian American race (OR = 0.72, 95% CI: 0.41–1.26) and Other race (OR = 0.43, 95% CI: 0.16–1.14) while Other vs. Caucasian race was associated with 68% higher NI odds (OR = 1.68, 95% CI: 0.68–4.13).

### 3.2. High Toxic Stress Is Associated with Higher NI; Relationship Varies by Mastery Level and Age

**Figure 2:**
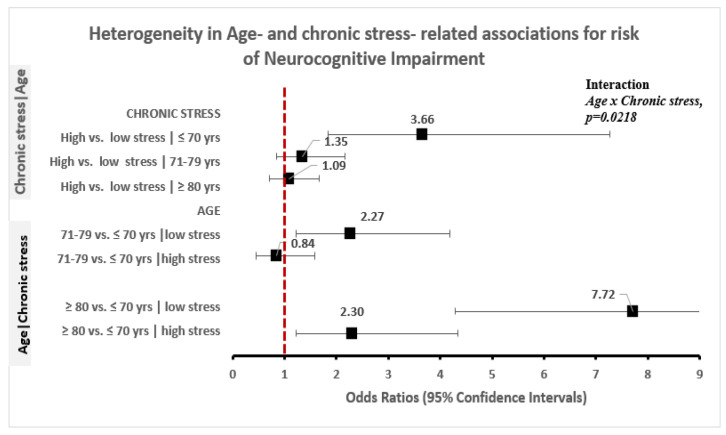
Heterogeneity in age and chronic stress-related associations for risk of neurocognitive impairment.

The odds of NI, was elevated for adults that reported high vs. low levels of chronic stress (OR = 1.88, 95% CI: 1.43–2.49), the experience of one or more vs. no everyday discrimination (OR = 4.05, 95% CI: 2.61–6.30) and having high vs. low perceived constraints (OR = 2.91, 95% CI: 2.03–4.17) in unadjusted models (Table 3). The magnitude of these associations was down modulated, but remained statistically robust, with sequential adjustment for socio-demographic factors, lifestyle factors, comorbidity, and toxic stress (Model 3) and RPF (Model 4, Table 3). However, the magnitude of discrimination-related differences in risk of NI varied according to level of mastery (discrimination × mastery, *p* = 0.0297; Figure 1). Specifically, the experience of any vs. no discrimination was associated with twice the odds of NI (OR = 1.95, 95% CI: 1.09–3.46) among adults with low mastery and 5.4 times the odds of NI (OR = 5.42, 95% CI: 2.99–9.83) among adults with high mastery. Furthermore, low vs. high stress associated risk of NI varied according to age (chronic stress × age, *p* = 0.022; Figure 2) and with stress-related elevation of NI risk evident among older Americans aged ≤ 70 years (OR = 3.66, 95% CI: 1.84–7.27) but not among individuals aged 71–79 years (OR = 1.35, 95% CI: 0.85–2.16) or ≥ 80 years old (OR = 1.09, 95% CI: 0.75–1.67).

### 3.3. Low RPF Is Associated with NI, But Relationship Varies by Race and the Experience of Discrimination

**Figure 3:**
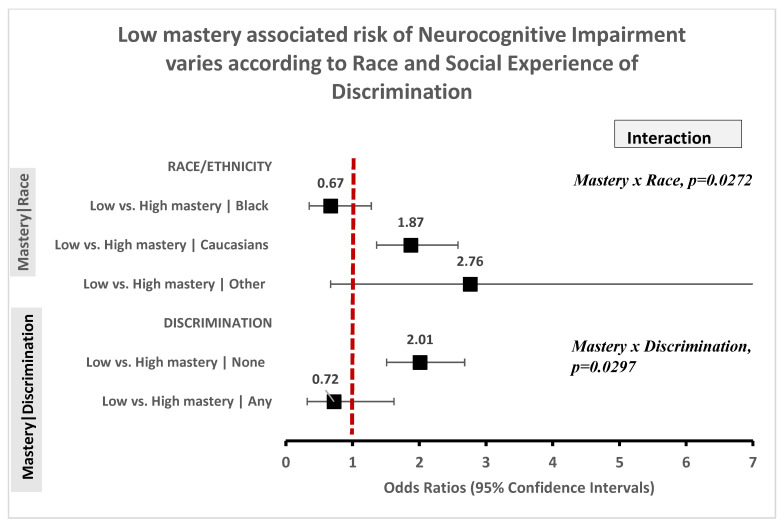
Low mastery associated risk of Neurocognitive impairment varies within strata of race, and social experience of discrimination.

Low vs. high levels of RPF were consistently associated with higher odds of NI (OR = 1.93–2.40, 95% CI: 1.46–3.38) in unadjusted models. This association was down modulated with adjustment for confounding covariates but the odds of NI remained elevated for adults reporting low vs. high levels of perceived global and domain specific mastery (OR = 1.70–2.02, 95% CI: 1.31–2.85) and for individuals reporting low vs. high positive social support from key relationships (OR = 1.45, 95% CI: 0.95–2.21; Table 3). However, the relationship between global mastery and NI differed according to the race/ethnicity of older Americans (mastery x race, *p* = 0.027; Figure 3). Specifically, low vs. high mastery was associated with higher odds of NI among Whites (OR = 1.87, 95% CI: 1.36–2.58) and among Americans of Other race (OR = 2.76, 95% CI: 0.67–11.40) but not among Blacks (OR = 0.67, 95% CI: 0.35–1.28). Likewise, low vs. high mastery was associated with 100% increase in odds of NI (OR = 2.01 95% CI: 1.51–2.68) among older Americans without the social experience of everyday discrimination but no relationship was evident between mastery and NI (OR = 0.72, 95% CI: 0.32–1.62) among Americans that reported one or more experiences of everyday discrimination.

### 3.4. Increasing Age Is Associated with Higher Risk of NI; Relationship Varies by Level of Stress

Increasing age was also associated with higher odds of NI (OR = 1.36–4.73, 95% CI: 0.86–7.43) in unadjusted models. This association was down modulated with adjustment for confounders, but the odds of NI remained elevated for adults ≥ 80 years old compared to adults aged ≤ 70 years (OR = 4.34, 95% CI: 2.74–6.87; Supplementary Table S2). Of note, age-related differences in NI odds varied according to high vs. low chronic stress (chronic stress × age, *p* = 0.022; Figure 2). Specifically, among adults with low levels of chronic stress, the odds of NI was respectively twice (OR = 2.27, 95% CI: 1.23–4.19) and nearly eight (OR = 7.72, 95% CI: 4.28–13.91) times as high for adults 71–79 years and ≥80 years old compared to adults aged ≤70 years. Among older Americans with high chronic stress on the other hand, the odds of NI was comparable for adults 71–79 vs. ≤70 years old (OR = 0.84, 95% CI: 0.45–1.58), and was elevated but to a lower degree for adult Americans ≥80 vs. ≤70 years old (OR = 2.30, 95% CI: 1.22–4.34).

### 3.5. Other Factors Associated with Increased Risk of NI

Among other factors, lower education status and having comorbid HD, T2DM, or stroke were each associated with increased risk of NI after controlling for TS, RPF, and potential confounders (Supplementary Table S2).

## 4. Discussion

In this population-based cohort study of 6317 older Americans, we found that higher levels of TS (chronic stress and discrimination) and lower levels of RPF, e.g., mastery, were associated with an increased risk for NI. Furthermore, we found novel empirical evidence that in the presence of discrimination, the benefit of high mastery for cognitive reserve is muted. These findings were consistent with our study hypothesis and align with prior research among adults with trauma that associated the presence of post-traumatic stress disorder (PTSD) with a greater NI risk [47]. These findings are also in line with reported higher perceived stress-related worse cognitive function and more rapid cognitive decline in a community-based study of Black and White American adults [48]. Extant literature relating childhood adversity or trauma exposure with neuropsychiatric morbidity—specifically depression, anxiety [49] and adverse cognitive function in adulthood [50] support the reported association. Furthermore, mechanistic insight underlying the findings of this large epidemiological study—specifically that psychological stress is associated with detectable cellular changes in regions of the hippocampus, decreased proliferation of neurons in the dentate gyrus, and with loss of hippocampal volume resulting in atrophy and cognitive deficits have been delineated [51,52,53,54,55,56,57].

Our findings on the elevated risk of NI due to increasing age, low education status and the presence of comorbid HD, T2DM or stroke confirm previous observations that advanced chronologic age [58], low educational attainment [24,59] and the presence of metabolic chronic disease [60,61,62] are important determinants of NI. In line with some [24,48] but not all prior studies, we found limited evidence of substantial race-related differences in rates of NI in this diverse sample of older American adults. Data from this study suggests that psychosocial adversity—i.e., TS- and RPF-associated NI risk was generally stronger than race-related differences in NI risk. While overall difference in NI risk by race was limited, our study expands the scope of knowledge pertaining to how disparate social experiences by race may accentuate disparity in cognitive function among older Americans because African-American vs. White or Other race-associated disadvantage in NI persisted among Americans that achieved higher levels of mastery. Of note, mastery is an indicator of intrinsic capacity for control, self-efficacy, competency or demonstrated effectiveness at achieving personal and social goals. Over-represented in the high end of mastery would be older Americans of higher socioeconomic status (education, occupation, income) and by extension, those with above average access to health care resources and the wherewithal to benefit from self-directed health agency to counteract a health risk. Therefore, high mastery status is expected to be neuro-protective regardless of race.

Unlike older Americans of White or Other race, the expected benefit of achieving high mastery for cognitive reserve, is muted or absent for older African American adults in this study. This observation is similar to previously reported higher levels of allostasis for Black and Mexican Americans relative to White Americans with a college degree or higher, whereas allostasis was similar across race groups among adults with low educational achievement in the same study [63]. Race is a well described social determinant of health and overall wellbeing in the US [64,65,66]. Prior data shows that Black Americans of higher educational status report high frequency of experienced micro-aggression and work-place discrimination and more frequently report being in jobs below their qualification level [67]. Both the nature and frequency of everyday discrimination varies according to race, with African Americans more frequently on the receiving end of the most insidious forms of discrimination in occupational and social interactions—whether in healthcare, educational, financial, law enforcement, and judicial systems [67,68]. Common experiences of discrimination related psychosocial stressors, such as receiving less respect, poorer services, being considered unintelligent, being perceived as threatening, not receiving benefit of the doubt and numerous other forms of adverse social experiences are more frequent among individuals of African American descent [69,70,71], do not change substantially by objective mastery level and mediate higher risk of adverse physical [69,72,73,74,75], and reproductive health outcomes [76,77,78]. Our data suggests that the differential amplification of psychosocial adversity—likely due to status inconsistency for African Americans with high mastery, overwhelms the expected neuroprotective benefit of mastery for high-mastery African Americans relative to high-mastery Americans of White or Other race.

The implementation of a large nationally representative study using rigorous analytic approaches adjusted for multiple confounders is an important strength of this study. An additional strength and novel contribution of the present study lies in the evaluation of multiple indicators of TS and resiliency as proxies for social experiences that affect cognitive aging. Limitations of the present study include its cross-sectional design which limits causal inference due to potential for residual confounding by unmeasured factors (e.g., additional aspects of socioeconomic status, physical activity) and inability to infer temporal sequence. Further limitation lies in low statistical power to evaluate heterogeneity in relationship of social determinants to NI particularly within the stratum of Other race.

## 5. Conclusions

Maintaining cognitive reserve is crucial for promoting healthy life span and acceptable quality of life in advanced age [79,80,81]. This study provided empirical evidence that high psychosocial adversity and low levels of RPF are important social determinants of NI and impaired cognitive reserve in a diverse sample of older US adults. African American race was associated with cognitive disadvantage, but only in the status inconsistent context of high mastery. Regardless of race, the benefit of high mastery for cognitive reserve among older Americans was muted among those that reported experiences of discrimination. Therefore, policy interventions that decrease psychosocial stress and opportunities that enhance social equity are needed to promote healthy cognitive aging regardless of race. However, specific social policies/interventions to mitigate psychosocial adversity associated cognitive impairment must be tailored by race to maximize its effectiveness.

## Figures and Tables

**Table 1:** Demographic characteristics of older American adults enrolled in the Health and Retirement Study (HRS) 2012–2014 sample by cognitive function status.

Characteristic	All (*N* = 6317)	Normal (*N* = 6019)	Impaired (*N* = 298)	*p*-Value
	*N* (%)	*N* (%)	*N* (%)	
Age: mean (SD)	73.9 (6.7)	73.7 (6.5)	78.7 (8.1)	
Age categories (years)				<0.0001
≤70	2076 (32.9)	2025 (33.6)	51 (17.1)	
71–79	3055 (48.4)	2942 (48.9)	113 (37.9)	
>80	1186 (18.7)	1052 (17.5)	132 (45.0)	
Sex: Female	3764 (59.6)	3601 (59.8)	163 (54.7)	0.0782
Marital Status				0.0002
Never married	177 (02.8)	167 (02.8)	10 (03.3)	
Married/partnered	3910 (61.9)	3761 (62.5)	149 (50.0)	
Separated/Divorced	672 (10.6)	633 (10.5)	39 (13.1)	
Widowed	1558 (24.7)	1458 (24.2)	100 (33.6)	
Education				<0.0001
Less than High School/GED	1326 (21.0)	1203 (20.0)	123 (41.3)	
High-school graduate	2062 (32.6)	1979 (32.9)	83 (27.8)	
Some college and above	2927 (46.4)	2835 (47.1)	92 (30.9)	
Race				0.0414
White/Caucasian	5217 (82.6)	4987 (82.8)	230 (77.2)	
Black/AA	815 (12.9)	765 (12.7)	50 (16.8)	
Other	285 (04.5)	267 (04.4)	18 (06.0)	
Have Health Insurance	6081 (98.1)	5810 (98.1)	271 (97.1)	0.2474
Ever smoked	3459 (55.2)	3299 (55.2)	160 (54.2)	0.7745
Current alcohol use	3209 (50.8)	3120 (51.8)	89 (29.9)	<0.0001
No. of comorbidities ever had				<0.0001
None	424 (06.7)	410 (06.8)	14 (04.7)	
One	1096 (17.3)	1061 (17.6)	35 (11.7)	
Two	1719 (27.2)	1665 (27.7)	54 (18.1)	
Three or more	3077 (48.7)	2882 (47.9)	195 (65.4)	
Diagnosis of HD, T2DM or Stroke	3177 (50.2)	2973 (49.4)	204 (68.5)	<0.0001
Measures of Toxic Stress				
Chronic stressors				0.005
Low	4542 (71.9)	4349 (72.2)	193 (64.8)	
High	1775 (28.1)	1670 (27.8)	105 (35.2)	
Everyday discrimination				<0.0001
Zero	5888 (94.3)	5638 (94.8)	250 (84.5)	
One or more	357 (05.7)	311 (05.2)	46 (15.5)	
Perceived constraints				<0.0001
Low	4262 (67.8)	4122 (68.9)	140 (47.0)	
High	2021 (32.2)	1863 (31.1)	158 (53.0)	
Measures of resilience				
Personal mastery				<0.0001
Low	2206 (34.9)	2049 (34.0)	157 (52.7)	
High	4111 (65.1)	3970 (66.0)	141 (47.3)	
Control over Health				<0.0001
Low	2794 (45.6)	2624 (44.9)	170 (60.1)	
High	3329 (54.4)	3216 (55.1)	113 (39.9)	
Control over finances				<0.0001
Low	2375 (38.1)	2224 (37.4)	151 (51.7)	
High	3867 (61.9)	3726 (62.6)	141 (48.3)	
Control over social life				<0.0001
Low	1892 (30.3)	1748 (29.4)	144 (49.7)	
High	4345 (69.7)	4199 (70.6)	146 (50.3)	
Positive Social Support domains				
Immediate Family (Spouse & children)				0.0247
Low social support	2314 (38.6)	2188 (38.3)	126 (45.0)	
High social support	3678 (61.4)	3524 (61.7)	154 (55.2)	
Extended Family (Others & friends)				0.0095
Low social support	1473 (23.6)	1388 (23.3)	85 (30.0)	
High social support	4759 (76.4)	4561 (76.7)	198 (70.0)	
All Relationship groups combined				<0.0001
Low social support	841 (13.3)	772 (12.9)	69 (23.1)	
High social support	5462 (86.7)	5233 (87.1)	229 (76.9)	

SD = Standard Deviation. 02 missing education; 116 missing health insurance; 46 missing smoking status; 1 missing alcohol consumption; 72 missing measures of everyday discrimination; 34 missing perceived constraints; 194 missing information on control over health; 80 missing information on control over social life; 75 missing information on financial control; 325 missing information on positive social support from immediate family; 85 missing information on positive social support from extended family; 14 missing information on positive social support from all relationship groups combined.

**Table 2:** Distribution of Toxic Stress and Resilience promoting factors among Older American adults enrolled in the HRS 2012–2014 sample at baseline by Race.

Characteristic	All (*N* = 6317)	White/Caucasian (*N* = 5217)	Black/African American (*N* = 815)	Other (*N* = 285)	*p*-Value
Dimensions of Toxic Stress	*N* (%)	*N* (%)		*N* (%)	
Chronic stressors					0.0084
Low	4542 (71.9)	3792 (72.7)	560 (68.7)	190 (66.7)	
High	1775 (28.1)	1425 (27.3)	255 (31.3)	95 (33.3)	
Everyday discrimination					<0.0001
Zero	5888 (94.3)	4925 (95.3)	716 (90.2)	247 (87.2)	
One or more	357 (5.8)	244 (4.7)	78 (9.8)	35 (12.8)	
Perceived constraints					0.0009
Low	4262 (67.8)	3538 (68.2)	561 (69.3)	163 (57.8)	
High	2021 (32.2)	1653 (31.8)	249 (30.7)	119 (42.2)	
Measures of resilience					
Personal mastery					0.9508
Low	2206 (34.9)	1820 (34.9)	288 (35.3)	98 (34.4)	
High	4111 (65.1)	3397 (65.1)	527 (64.7)	187 (65.6)	
Control over Health					0.2371
Low	2794 (45.6)	2330 (46.0)	351 (45.2)	113 (40.8)	
High	3329 (54.4)	2740 (54.0)	425 (54.8)	164 (50.2)	
Control over finances					0.1666
Low	2375 (38.1)	1989 (38.5)	280 (35.0)	106 (37.7)	
High	3867 (61.9)	3173 (61.5)	519 (65.0)	175 (62.3)	
Control over social life					0.0155
Low	1892 (30.3)	1594 (30.9)	207 (26.0)	91 (32.3)	
High	4345 (69.7)	3565 (69.1)	589 (74.0)	191 (67.7)	
Positive Social Support (All Relationship groups combined)					0.3237
Low social support	841 (13.3)	681 (13.1)	115 (14.2)	45 (15.8)	
High social support	5462 (86.7)	4525 (86.9)	697 (85.8)	240 (84.2)	
Neurocognitive impairment					0.0414
Normal	6019 (95.3)	4987 (95.6)	765 (93.9)	267 (93.7)	
Impaired	298 (4.7)	230 (4.4)	50 (6.1)	18 (6.3)	

72 missing measures of everyday discrimination; 34 missing perceived constraints; 194 missing information on control over health; 80 missing information on control over social life; 75 missing information on financial control; 325 missing information on positive social support from immediate family; 85 missing information on positive social support from extended family; 14 missing information on positive social support from all relationship groups combined.

**Table 3:** Race, Toxic Stress, and Resilience promoting factors in relation to risk for Neurocognitive impairment among older American adults enrolled in the HRS 2012–2014.

Variable	*n*/*N*	Model 1 (Crude) *	Model 2 ^ɤ^	Model 3 ^α^	Model 4 ^†^
Race		OR (95% CI)	OR (95% CI)	OR (95% CI)	OR (95% CI)
Black (AA) vs. Caucasian	50/815	**1.76 (1.21, 2.56)**	1.33 (0.89, 1.99)	1.26 (0.82, 1.94)	1.32 (0.87, 2.00)
Other vs. Caucasian	18/285	**2.09 (1.18, 3.72)**	1.63 (0.95, 2.82)	1.45 (0.87, 2.43)	1.59 (0.93, 2.72)
Toxic stress indicators					
Everyday discrimination					
One or more experiences vs. None	46/357	**4.05 (2.61, 6.30)**		**3.31(2.12, 5.19)**	
Chronic stressors					
High vs. low chronic stress	105/1775	**1.88 (1.43, 2.49)**		**1.88 (1.42, 2.48)**	
Perceived constraints					
High vs. Low	158/2021	**2.91 (2.03, 4.17)**		**2.16 (1.52, 3.07)**	
Resilience indicators					
Perceived Mastery					
Low vs. High global mastery	157/2206	**2.38 (1.78, 3.20)**			**1.85 (1.38, 2.48)**
Positive social support from all groups					
Low vs. High	69/841	**1.89 (1.36, 2.62)**			1.45 (0.95, 2.21)
Domain-specific mastery					
Low vs. High control over health	170/2794	**2.04 (1.54, 2.70)**			**1.70 (1.31, 2.21)**
Low vs. High control over finances	151/2375	**1.93 (1.46, 2.57)**			**1.96 (1.44, 2.67)**
Low vs. High Social life	144/1892	**2.40 (1.70, 3.38)**			**2.02 (1.43, 2.85)**

OR (95% CI): Odds Ratios (95% Confidence Intervals); Bold indicates *p*-value < 0.05; * Model 1 are crude models. Models 2–4 are adjusted models. ^ɤ^ Model 2- adjusts for race and demographic factors, age, sex, education, alcohol consumption, smoking, BMI, and comorbidity due to Diabetes, Heart diseases and Stroke. ^α^ Model 3 adjusts for race and demographic factors, age, sex, education, alcohol consumption, smoking, BMI, and comorbidity due to Diabetes, Heart diseases and Stroke plus Toxic stress measures. ^†^ Model 4 adjusts for race and demographic factors, age, sex, education, alcohol consumption, smoking, BMI and comorbidity due to Diabetes, Heart diseases and Stroke plus resilience indicators. Measures of toxic stress and indicators of resilience were not mutually adjusted for one another in any multivariable models.

## Data Availability

Publicly available datasets were analyzed in this study. This data can be found here: https://hrs.isr.umich.edu/data-products/access-to-public-data.

## Supplementary Materials

The following are available online at https://www.mdpi.com/1660-4601/18/3/1358/s1, Table S1: Demographic characteristics of older American adults enrolled in the HRS 2012-14 sample by Race, Table S2: Other factors in relation to risk for Neurocognitive impairment among older adults from HRS 2012-14, and Table S3: The relationship between Race and Neurocognitive Impairment with or without adjustment for Toxic Stress or Resilience promoting factors.
