# Supplementary material for: The Relationship of Race, Psychosocial Stress and Resiliency Indicators to Neurocognitive Impairment among Older Americans Enrolled in the Health and Retirement Survey: A Cross-Sectional Study
Source: Int J Environ Res Public Health. 2021 Feb 2;18(3):1358. doi: 10.3390/ijerph18031358 (PMC7908633; doi:10.3390/ijerph18031358)
Supplement: Supplementary file 1 [file ijerph-18-01358-s001.pdf]

Assessing the impact of race, psychosocial stress and resiliency indicators on neurocognitive impairment: A cross-sectional study of older Americans enrolled in the Health and Retirement Survey

Supplementary Tables

Supplementary Table 1: Demographic characteristics of older American adults enrolled in the HRS 2012-14 sample by Race.

	All (N=6317)	White/ Caucasian (N=5217)	Black / African American (N=815)	Other (N=285)	
**Characteristic**	**N (%)**	**N (%)**	**N (%)**	**N (%)**	**p-value**
**Age: mean(SD)**	73.9 (6.7)	74.2 (6.7)	72.6 (6.3)	72.2 (6.0)	
**Age categories (years)**					< 0.0001
<=70	2076 (32.9)	1615 (31.0)	332 (40.7)	129 (45.3)	
71-79	3055 (48.4)	2555 (49.0)	382 (46.9)	118 (41.4)	
>80	1186 (18.8)	1047 (20.1)	101 (12.4)	38 (13.3)	
**Sex**					0.003
Female	3764 (59.6)	3077 (59.0)	528 (64.8)	159 (55.8)	
**Marital Status**					< 0.0001
Never married	177 (02.8)	113 (2.2)	48 (5.9)	16 (5.6)	
Married/ partnered	3910 (61.9)	3381 (64.8)	355 (43.6)	174 (61.0)	
Separated/Divorced	672 (10.6)	475 (9.1)	159 (19.5)	38 (13.3)	
Widowed	1558 (24.7)	1248 (23.9)	253 (31.0)	57 (20.0)	
**Education**					< 0.0001
Less than High School/GED	1326 (21.0)	961 (18.4)	255 (31.3)	110 (38.6)	
High-school graduate	2062 (32.6)	1751 (33.6)	255 (31.3)	56 (19.6)	
Some college and above	2927 (46.4)	2504 (48.0)	304 (37.4)	119 (41.8)	
**Health Insurance**					0.0003
Yes	6081 (98.1)	5033 (98.3)	783 (97.6)	265 (95.0)	
**Ever smoked**					0.6196
Yes	3459 (54.8)	2851 (54.6)	457 (56.1)	151 (53.0)	
**Current alcohol use**					< 0.0001
Yes	3209 (50.8)	2802 (53.7)	296 (36.3)	111 (38.9)	
**No. of comorbidities ever had**					< 0.0001
None	424 (06.7)	360 (6.9)	35 (4.3)	29 (10.2)	
One	1096 (17.3)	939 (18.0)	105 (12.9)	52 (18.2)	
Two	1719 (27.2)	1415 (27.1)	241 (29.6)	63 (22.1)	
Three or more	3077 (48.7)	2502 (48.0)	434 (53.2)	141 (49.5)	
**Diagnosis of HD, T2DM or Stroke**					< 0.0006
Yes	3177 (50.2)	2566 (49.2)	454 (55.7)	157 (55.1)	
**Ever had High blood pressure**					< 0.0001
Yes	4317 (68.5)	3443 (66.1)	678 (83.3)	196 (69.0)	
**BMI**					< 0.0001
BMI < 18.5 kg/m^2 (Underweight)	101 (1.6)	85 (1.6)	10 (1.2)	6 (2.1)	
BMI 18.5-24 kg/m^2 (Normal weight)	1736 (27.5)	1508 (28.9)	145 (17.8)	83 (29.1)	
BMI 25-29 kg/m^2 (Overweight)	2390 (37.8)	1976 (37.9)	305 (37.4)	109 (38.3)	
BMI ≥30 kg/m^2 (Obese)	2090 (33.1)	1648 (31.6)	355 (43.6)	87 (30.5)	

SD=Standard Deviation
Note: 02 missing education; 116 missing health insurance; 46 missing smoking status; 1 missing alcohol consumption; 14 missing High blood pressure.

Supplementary Table 2: Other factors in relation to risk for Neurocognitive impairment among older adults from HRS 2012-14.

Variable	n/N	Model 1 (Crude)*	Model 2[ˠ]	Model 3[α]	Model 4[†]
**Age categories (years)**		**OR (95% CI)**	**OR (95% CI)**	**OR (95% CI)**	**OR (95% CI)**
71-79 vs <=70	113/3055	**1.36 (0.86, 2.16)**	1.20 (0.76, 1.89)	1.25 (0.81, 1.95)	1.22 (0.78, 1.93)
>80 vs <=70	134/1186	**4.73 (3.01, 7.43)**	**4.0 (2.50, 6.50)**	**4.34 (2.74, 6.87)**	**3.82 (2.35, 6.21)**
**Education**					
Less than High School vs College and above	123/1326	**3.08 (2.22, 4.26)**	**2.07 (1.55, 2.78)**	**1.94 (1.43, 2.64)**	**2.04 (1.51, 2.75)**
High School vs College and above	83/2062	1.08 (0.75, 1.56)	0.86 (0.59, 1.24)	0.84 (0.58, 1.22)	0.85 (0.58, 1.24)
**Marital Status**					
Never married vs Married	10/177	1.81 (0.72, 4.54)	1.79 (0.71, 4.50)	1.64 (0.66, 4.05)	1.75 (0.69, 4.44)
Separated/divorced vs Married	39/672	**1.62 (1.10, 2.39)**	**1.65 (1.09, 2.49)**	**1.63 (1.09, 2.43)**	**1.67 (1.11, 2.51)**
Widowed vs Married	100/1558	**2.03 (1.42, 2.92)**	1.23 (0.81, 1.87)	1.23 (0.82, 1.84)	1.21 (0.80, 1.83)
**Sex**					
Male vs Female	135/2553	1.17 (0.91, 1.50)	**1.48 (1.13, 1.93)**	**1.44 (1.11, 1.87)**	**1.51 (1.17, 1.95)**
**Body Mass Index**					
Underweight vs normal	7/101	1.77 (0.81, 3.86)	1.59 (0.62, 4.07)	1.35 (0.42, 4.28)	1.48 (0.56, 3.93)
Overweight vs normal	106/2333	0.83 (0.58, 1.18)	0.86 (0.60, 1.22)	0.86 (0.61, 1.21)	0.85 (0.60,1.21)
Obese vs normal	89/2098	0.87 (0.59, 1.28)	0.89 (0.62, 1.29)	0.87 (0.60, 1.27)	0.88 (0.61, 1.27)
**Alcohol consumption**					
No vs Yes	209/3111	**2.59 (1.88, 3.57)**	**1.98 (1.43, 2.75)**	**1.97 (1.44, 2.69)**	**1.94 (1.40, 2.68)**
**Cigarette smoking**					
Yes vs No	161/3473	0.96 (0.76, 1.22)	0.96 (0.73, 1.27)	0.97 (0.73, 1.31)	0.94 (0.71, 1.25)
**Health conditions**					
Comorbid HD, Diabetes or Stroke					
**Yes vs No**	205/3183	**2.63 (1.84, 3.76)**	**2.04 (1.40, 2.98)**	**1.99 (1.37, 2.89)**	**1.98 (1.37, 2.86)**

Notes: Odds Ratios (95% Confidence Intervals); Bold indicates p-value<0.05.*Model 1 are crude models. Models 2-4 are adjusted models.
ˠ Model 2 adjusts for demographic factors; age, sex, race, education, alcohol consumption, smoking, BMI and comorbidity due to Diabetes, Heart diseases and Stroke.
α Model 3 adjusts for demographic factors; age, sex, race, education, alcohol consumption, smoking, BMI and comorbidity due to Diabetes, Heart diseases and Stroke plus Toxic stress measures.
† Model 4 adjusts for demographic factors- age, sex, race, education, alcohol consumption, smoking, BMI and comorbidity due to Diabetes, Heart diseases plus Stroke and indicators of resilience.

Supplementary Table 3: The relationship between Race and Neurocognitive Impairment with or without adjustment for Toxic Stress or Resilience promoting factors.

			Model 1 (Crude)*	Model 5‡	
**Characteristic**	**Adjusted for**	**Association**	**OR (95% CI)**	**OR (95% CI)**	**p-value (Interaction)**
					Race*Discrimination
Race	Discrimination	Black (AA) vs Caucasian	**1.76 (1.21, 2.56)**	1.33 (0.74, 2.40)	0.3653
		Other vs Caucasian	**2.09 (1.18, 3.72)**	1.78 (0.89, 3.56)	
		Black (AA) vs Other	0.84 (0.42, 1.68)	0.75 (0.33, 1.69)	
					Race*Mastery
Race	Mastery	Black (AA) vs Caucasian	**1.76 (1.21, 2.56)**	1.20 (0.79, 1.82)	0.0272
		Other vs Caucasian	**2.09 (1.18, 3.72)**	1.38 (0.77, 2.47)	
		Black (AA) vs Other	0.84 (0.42, 1.68)	0.87 (0.44, 1.72)	
**Other predictors**					
					Discrimination*Mastery
Discrimination	Mastery	One or more experiences vs None	**4.05 (2.61, 6.30)**	**3.25 (2.14, 4.92)**	0.0297
					Mastery*Age
Mastery	Age	Low vs High global mastery	**2.38 (1.78, 3.20)**	**1.73 (1.28, 2.34)**	0.5851
					Discrimination*Age
Discrimination	Age	One or more experiences vs None	**4.05 (2.61, 6.30)**	**2.76 (1.87, 4.08)**	0.1379
					Chronic stress*Age
Chronic stress	Age	High vs low chronic stress	**1.88 (1.43, 2.49)**	**1.75 (1.28, 2.41)**	0.0218

Notes: OR (95%CI): Odds Ratios (95% Confidence Intervals); Bold indicates p-value<0.05; *Model 1 are crude models.
‡Models 5 adjust for race and demographic factors- age, sex, education, alcohol consumption, smoking, BMI and comorbidity due to Diabetes, Heart diseases plus Stroke and resilience indicators. Interaction terms have been added as well.
